# Machine Learning-Based Prediction of Lymph Node Metastasis Among Osteosarcoma Patients

**DOI:** 10.3389/fonc.2022.797103

**Published:** 2022-04-20

**Authors:** Wenle Li, Yafeng Liu, Wencai Liu, Zhi-Ri Tang, Shengtao Dong, Wanying Li, Kai Zhang, Chan Xu, Zhaohui Hu, Haosheng Wang, Zhi Lei, Qiang Liu, Chunxue Guo, Chengliang Yin

**Affiliations:** ^1^ Department of Orthopedics, Xianyang Central Hospital, Xianyang, China; ^2^ Clinical Medical Research Center, Xianyang Central Hospital, Xianyang, China; ^3^ School of Medicine, Anhui University of Science and Technology, Huainan, China; ^4^ Affiliated Cancer Hospital, Anhui University of Science and Technology, Huainan, China; ^5^ Department of Orthopaedic Surgery, the First Affiliated Hospital of Nanchang University, Nanchang, China; ^6^ School of Physics and Technology, Wuhan University, Wuhan, China; ^7^ Department of Spine Surgery, Second Affiliated Hospital of Dalian Medical University, Dalian, China; ^8^ Department of Spine Surgery, Liuzhou People’s Hospital, Liuzhou, China; ^9^ Department of Orthopaedics, The Second Hospital of Jilin University, Changchun, China; ^10^ Chronic Disease Division, Luzhou Center for Dcontrol and Prevention, Luzhou, China; ^11^ Biostatistics Department, Hengpu Yinuo (Beijing) Technology Co., Ltd, Beijing, China; ^12^ Faculty of Medicine, Macau University of Science and Technology, Macau, Macau SAR, China

**Keywords:** osteosarcoma, lymph node metastasis, SEER, multicenter, machine learning algorithm, web calculator

## Abstract

**Background:**

Regional lymph node metastasis is a contributor for poor prognosis in osteosarcoma. However, studies on risk factors for predicting regional lymph node metastasis in osteosarcoma are scarce. This study aimed to develop and validate a model based on machine learning (ML) algorithms.

**Methods:**

A total of 1201 patients, with 1094 cases from the surveillance epidemiology and end results (SEER) (the training set) and 107 cases (the external validation set) admitted from four medical centers in China, was included in this study. Independent risk factors for the risk of lymph node metastasis were screened by the multifactorial logistic regression models. Six ML algorithms, including the logistic regression (LR), the gradient boosting machine (GBM), the extreme gradient boosting (XGBoost), the random forest (RF), the decision tree (DT), and the multilayer perceptron (MLP), were used to evaluate the risk of lymph node metastasis. The prediction model was developed based on the bestpredictive performance of ML algorithm and the performance of the model was evaluatedby the area under curve (AUC), prediction accuracy, sensitivity and specificity. A homemade online calculator was capable of estimating the probability of lymph node metastasis in individuals.

**Results:**

Of all included patients, 9.41% (113/1201) patients developed regional lymph node metastasis. ML prediction models were developed based on nine variables: age, tumor (T) stage, metastasis (M) stage, laterality, surgery, radiation, chemotherapy, bone metastases, and lung metastases. In multivariate logistic regression analysis, T and M stage, surgery, and chemotherapy were significantly associated with lymph node metastasis. In the six ML algorithms, XGB had the highest AUC (0.882) and was utilized to develop as prediction model. A homemade online calculator was capable of estimating the probability of CLNM in individuals.

**Conclusions:**

T and M stage, surgery and Chemotherapy are independent risk factors for predicting lymph node metastasis among osteosarcoma patients. XGB algorithm has the best predictive performance, and the online risk calculator can help clinicians to identify the risk probability of lymph node metastasis among osteosarcoma patients.

## Introduction

Lymph node metastasis is one of the most common metastases and has important prognostic implications for many types of cancers (1, 2). Systemic metastatic cells, originated from primary cancer, spread through the blood and lymphatic system, and lymph nodes are usually the first organ to develop metastases. In various types of tumors, the presence of metastatic tumor cells in regional lymph nodes is one indicator for poor prognosis (3, 4).

Osteosarcoma, also called osteogenic sarcoma, is a common primary malignant bone tumor and is highly malignant and aggressive (5). Osteosarcoma patients often suffered from poor prognosis due to metastases, with the lung being the most common site of metastasis (6). Besides, patients also could be found to develop lymph node metastasis in the ipsilateral or contralateral limb (7). The incidence of lymph node metastasis in osteosarcoma was 1.4% to 2.3%. Although the probability of lymph node metastasis in osteosarcoma was not high, it was still of a high concern owing to its significant association with reduced 5-year survival outcome among osteosarcoma patients (8). Sheila Thampi et al. (9) found that the 5-year overall survival rates for patients with and without regional lymph node involvement were 10.9% and 54.3%, respectively, and regional lymph node involvement was an independent predictor of relative poorer survival prognosis. These results suggested that the focus on osteosarcoma lymph node metastasis should be emphasized in clinical practice, and it was of particular importance to develop a predictive model to stratify the risk of osteosarcoma lymph node metastasis at advance.

Machine learning, a new type of artificial intelligence, is beginning to be widely used in healthcare data analysis and is a powerful tool to improve clinical strategies (10–14). Machine learning algorithms can automatically learn from the input data to predict output values within acceptable accuracy and identify patterns and trends in the data (11–17). Therefore, this study aimed to develop models based on machine learning using clinical features to predict the risk of lymph node metastasis among osteosarcoma patients so that individual prevention strategies for osteosarcoma could be proposed to help clinicians to make therapeutic decisions. Thus, we hypothesized that an optimal model could be developed with the help of significant clinical features according to machine learning.

## Methods

### Study Population

Retrospective analysis of the SEER (Surveillance, Epidemiology, and End Results) database and data of patients admitted to the Second Affiliated Hospital of Jilin University, the Second Affiliated Hospital of Dalian Medical University, the Liuzhou People’s Hospital affiliated to Guangxi Medical University, and the Xianyang Central Hospital. SEER is an authoritative source for cancer statistics in the United States. The Surveillance, and it provides information on cancer statistics in an effort to reduce the cancer burden among the U.S. population. Patients were included if they had (1) pathologically confirmed primary osteosarcoma, (2) no concurrent malignancy, and (3) complete clinical information, including age, gender, race, first site, T and M stage, surgery, and chemotherapy. Patients were excluded if they had (1) no complete clinical information, (2) other primary neoplastic disease, and (3) unknown metastatic status. For multicenter data, the study was approved by the ethics review committee of four medical institutions in China, the Second Affiliated Hospital of Jilin University, the Second Affiliated Hospital of Dalian Medical University, Liuzhou People’s Hospital, and Xianyang Central Hospital (No. 2021-00-22) and was conducted in accordance with the guidelines of the Helsinki Declaration.

### Construction, Validation and Clinical Utility of a Web-Based Calculator

The cohort from the SEER database was included in the traininggroup and the cohort from the four medical centers was includedin the validation group. We compared the pathologicalcharacteristics of the training and validation group andanalyzed the risk factors for predicting lymph node metastasisby the univariate analysis. Subsequently, the multivariate logisticregression analysis was used to evaluate each variable, andindependent predictors associating with lymph node metastasiswere obtained. The independent predictors were included in sixmachine learning algorithms and the AUC was calculated toidentify the highest performing machine learning model. Meanwhile, A web-based calculator was capable of estimating the probability of lymph node metastasis in individuals.

### Statistical Analysis

The training group was extracted from the SEER database using SEER statistical software (version 8.3.6). All analyses were performed using R software (version 3.6.0). Continuous variables were represented as the median with interquartile range (IQR), while categorical variables were represented as numbers with proportions. Differences of two groups were compared by Wilcoxon rank-sum test for continuous variables, and categorical variables were evaluated using the Chi-Squared test or Fisher’s Exact test. Logistic regression analysis was used to analyze the relationship between various predictor variables (either categorical or continuous) and an outcome which is binary (dichotomous). Six ML algorithms, including the logistic regression (LR), the gradient boosting machine (GBM), the extreme gradient boosting (XGBoost), the random forest (RF), the decision tree (DT), and the multilayer perceptron (MLP), were used to evaluate the risk of lymph node metastasis. The prediction model was developed based on the best predictive performance of ML algorithm and the performance of the model was evaluated by the area under curve (AUC), prediction accuracy, sensitivity and specificity.Bilateral p-values < 0.05 were considered statistically significant.

## Results

### Comparisons Between Patients With and Without Lymph Node Metastases

The 1201 patients were divided into two groups according to the presence of lymph node metastases, and the differences between the two groups (lymph node metastases vs no lymph node metastases) in terms of age (P=0.01), T stage (P<0.001), M stage (,P<0.001), surgery (P<0.001), chemotherapy (P=0.005), bone metastases (P<0.001), and survival times (P<0.001) were statistically significant (**Table 1**). Meanwhile, fewer patients in surgery owned lymph node metastases than non-surgical patients (**Table 1**).

**Table 1:** Description of the study population according to the presence of lymph node metastases.

Variables	levels	Overall (N=1201)	lymph node metastases		*P*
			No (N=1088)	Yes* (N=113)	
Category [n(%)]	Multicenter	107 (8.9)	91 (8.4)	16 (14.2)	0.059
	SEER	1094 (91.1)	997 (91.6)	97 (85.8)	
Race [n(%)]	black	163 (13.6)	143 (13.1)	20 (17.7)	0.089
	other	216 (18.0)	190 (17.5)	26 (23.0)	
	white	822 (68.4)	755 (69.4)	67 (59.3)	
Age(years) [M(Q1, Q3)]		32.98 (24.08)	32.41 (23.70)	38.53 (26.89)	0.01
Sex [n(%)]	female	549 (45.7)	492 (45.2)	57 (50.4)	0.336
	male	652 (54.3)	596 (54.8)	56 (49.6)	
Primary site [n(%)]	Axis bone	313 (26.1)	278 (25.6)	35 (31.0)	0.241
	Limb bone	787 (65.5)	721 (66.3)	66 (58.4)	
	other	101 (8.4)	89 (8.2)	12 (10.6)	
Laterality [n(%)]	left	514 (42.8)	477 (43.8)	37 (32.7)	0.074
	Not a paired site	161 (13.4)	144 (13.2)	17 (15.0)	
	right	526 (43.8)	467 (42.9)	59 (52.2)	
Stage group [n(%)]	I	198 (16.5)	193 (17.7)	5 (4.4)	<0.001
	II	562 (46.8)	542 (49.8)	20 (17.7)	
	III	51 (4.2)	48 (4.4)	3 (2.7)	
	IV	278 (23.1)	211 (19.4)	67 (59.3)	
	UNK stage	112 (9.3)	94 (8.6)	18 (15.9)	
T [n(%)]	T1	420 (35.0)	395 (36.3)	25 (22.1)	<0.001
	T2	562 (46.8)	520 (47.8)	42 (37.2)	
	T3	40 (3.3)	34 (3.1)	6 (5.3)	
	TX	179 (14.9)	139 (12.8)	40 (35.4)	
M [n(%)]	M0	931 (77.5)	873 (80.2)	58 (51.3)	<0.001
	M1	270 (22.5)	215 (19.8)	55 (48.7)	
Surgery [n(%)]	No	225 (18.7)	175 (16.1)	50 (44.2)	<0.001
	Yes	976 (81.3)	913 (83.9)	63 (55.8)	
Radiation [n(%)]	No	1054 (87.8)	959 (88.1)	95 (84.1)	0.269
	Yes	147 (12.2)	129 (11.9)	18 (15.9)	
Chemotherapy [n(%)]	No	236 (19.7)	202 (18.6)	34 (30.1)	0.005
	Yes	965 (80.3)	886 (81.4)	79 (69.9)	
Bone metastases [n(%)]	No	1144 (95.3)	1045 (96.0)	99 (87.6)	<0.001
	Yes	57 (4.7)	43 (4.0)	14 (12.4)	
Survival times(month) [M(Q1, Q3)]		24 (12, 48)	25(13, 49)	16(7, 31)	<0.001

SEER, surveillance epidemiology and end results; T, stage; M, metastasis.

*indicates patients with unable to evaluate lymph node metastases were also included.

### Univariate and Multivariate Analysis of Potential Factors for Predicting Lymph Node Metastases

In the univariate analysis, age (P<0.05), survival time (P<0.01), laterality (P<0.05), T (P<0.001), M (P<0.001), surgery (P<0.001), chemotherapy (P<0.01), bone metastases (P<0.001), and lung metastases (P<0.001) were significantly associated with the occurrence of lymph node metastasis in osteosarcoma, while there were no significant differences in race, sex, primary site, and radiation between the two groups. The multiple logistic regression analysis including statistically significant differences in univariate factors showed that T (OR: 2.330, 95%CI: 1.265-4.292, P<0.01), M (OR: 3.182, 95%CI: 1.606-6.302, P<0.01), surgery (OR: 0.455, 95%CI: 0.267-0.774, P<0.01), chemotherapy (OR: 0.510, 95%CI: 0.287-0.906, P<0.05) were independent predictors of lymph node metastasis (**Table 2**).

**Table 2:** Single and multi-factor logistic regression analysis for the modeling group.

Variables	Univariate analysis		Multivariate analysis	
	OR (95% CI)	p value	OR (95% CI)	p value
Age(years)	1.010(1.002-1.018)	<0.05	0.997(0.986-1.008)	0.574
Survival time(month)	0.984(0.973-0.994)	<0.01	0.999(0.999-1.011)	0.835
Race				
White	Ref	Ref	Ref	Ref
Black	1.576(0.927-2.679)	0.093	/	/
Other	1.138(0.567-2.285)	0.716	/	/
Sex				
Male	Ref	Ref	Ref	Ref
Female	1.241(0.818-1.883)	0.311	/	/
Primary site				
Limb bones	Ref	Ref	Ref	Ref
Axis of a bone	1.143(0.905-2.286)	0.124	/	/
other	1.562(0.787-3.102)	0.203	/	/
Laterality				
Left	Ref	Ref	Ref	Ref
Right	1.615(1.015-2.572)	<0.05	1.559(0.955-2.547)	0.076
Other	1.625(0.865-3.052)	0.131	0.997(0.487-2.039)	0.992
T				
T1	Ref	Ref	Ref	Ref
T2	1.131(0.657-1.148)	0.657	0.948(0.537-1.675)	0.855
T3	2.787(0.984-7.892)	0.054	1.498(0.492-4.567)	0.477
TX	4.179(2.371-7.363)	<0.001	2.330(1.265-4.292)	<0.01
M				
M0	Ref	Ref	Ref	Ref
M1	3.842(2.505-5.892)	<0.001	3.182(1.606-6.302)	<0.01
Surgery				
No	Ref	Ref	Ref	Ref
Yes	0.250(0.162-.0387)	<0.001	0.455(0.267-0.774)	<0.01
Radiation				
No	Ref	Ref	Ref	Ref
Yes	1.634(0.947-2.821)	0.078	/	/
Chemotherapy				
No	Ref	Ref	Ref	Ref
Yes	0.491(0.312-0.771)	<0.01	0.510(0.287-0.906)	<0.05
Bone metastases				
No	Ref	Ref	Ref	Ref
Yes	3.378(1.707-6.682)	<0.001	1.371(0.631-1.655)	0.425
Lung metastases				
No	Ref	Ref	Ref	Ref
Yes	2.823(1.800-4.428)	<0.001	0.826(0.412-1.655)	0.589

OR, odds ratio; CI, confident interval; T, stage; M, metastasis.

### The Performance of Machine Learning Models

**Figure 1:**
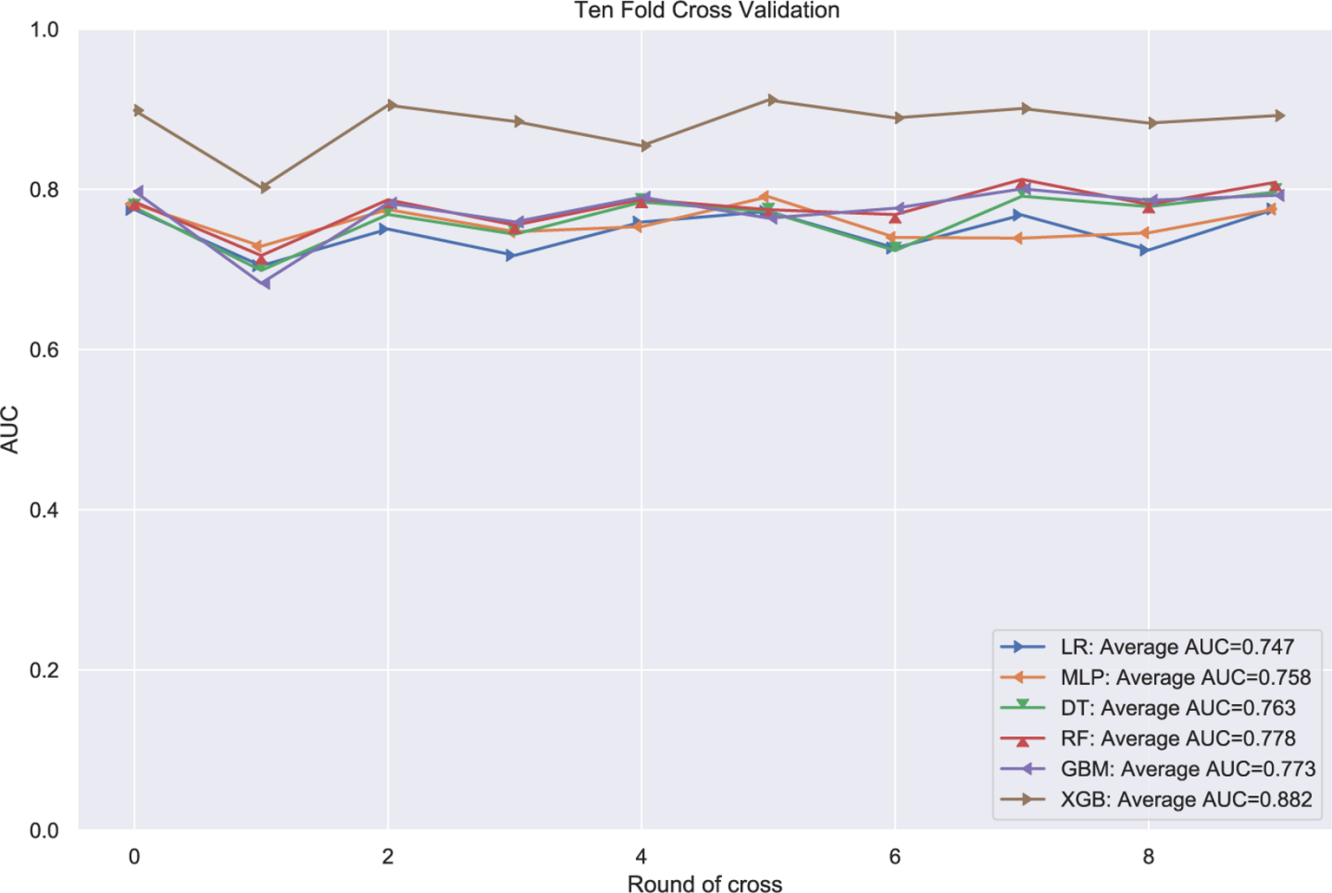
10-fold cross-validation of machine learning algorithms.

**Figure 2:**
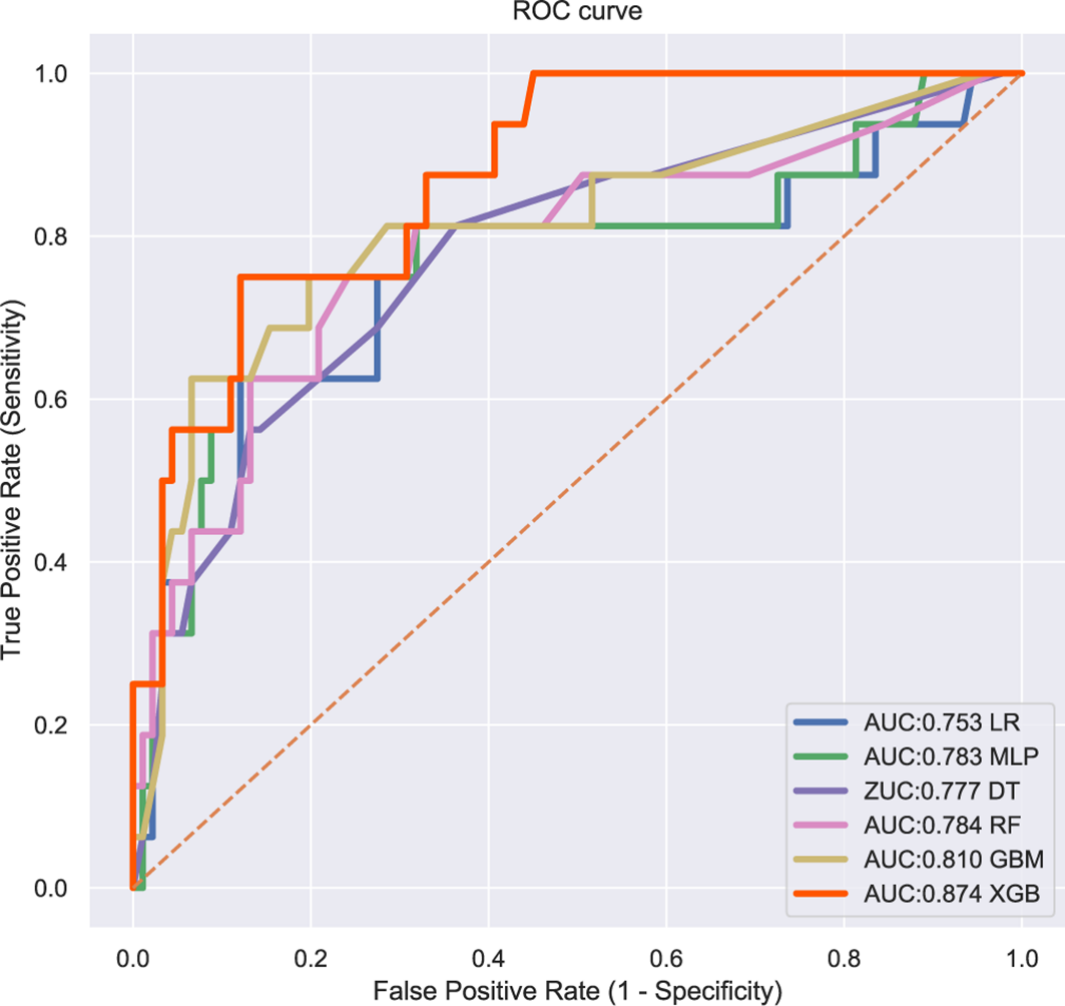
ROC curves for six ML algorithm models in predicting the risk of lung metastasis in osteosarcoma patients.

The patients in the training group were trained with 10-fold cross-validation, and the data set was divided into 10 parts, of which 9 parts were used for training and 1 part for testing on a rotating basis, and the final accuracy was averaged 10 times. The results (**Figure 1**) showed that the XGB model had the highest accuracy in predicting the risk of osteosarcoma lymph node metastasis occurrence with an AUC of 0.882. The results of external validation (**Figure 2**) also showed the best performance of the XGB model with an AUC of 0.874, a sensitivity of 0.750, a specificity of 0.868, and the accuracy of 0.851 (**Table 3**). Therefore, the XGB model was selected as the final prediction model in this study.

**Table 3:** Performance of the validation group models.

Models	AUC	Accuracy	Sensitivity	Specificity
LR	0.753	0.720	0.625	0.736
MLP	0.783	0.766	0.750	0.769
RF	0.777	0.729	0.750	0.725
DT	0.784	0.720	0.688	0.725
GBM	0.810	0.729	0.813	0.714
XGB	0.874	0.851	0.750	0.868

AUC, area under the curve; LR, logistic regression; MLP, multilayer perceptron; RF, random forest; DT, the decision tree; GBM, gradient boosting machine; XGBoost, extreme gradient boosting.

### Relative Importance of Variables in Machine Learning Algorithms

**Figure 3:**
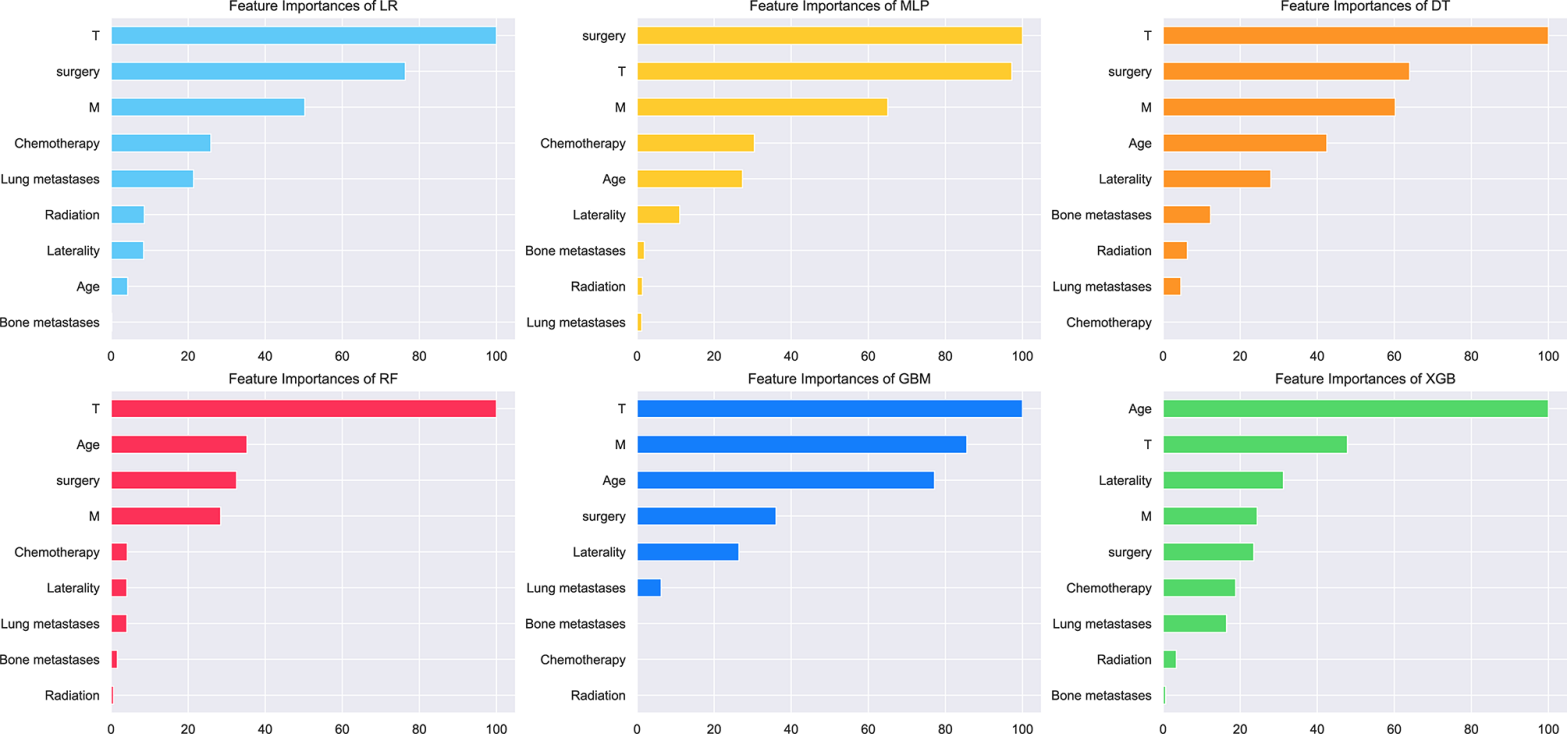
Relative importance ranking of features in six ML algorithms for predicting lymph node metastasis.

The relative importance of variables in each ML algorithm for predicting osteosarcoma lymph node metastasis is shown in **Figure 3**, and the overall trend was as follows: although the importance of variables in these ML algorithms varied slightly, they included T stage, M stage, age, surgery, and chemotherapy ranked in the top five. In contrast, Radiation, bone metastases, and lung metastases did not contribute much to the prediction of the risk of lymph node metastasis occurrence in osteosarcoma. The XGB model performed best with the following variables in descending order of importance: age, T stage, laterality, M stage, surgery, chemotherapy, lung metastases, radiation, and bone metastases.

### Web-Based Calculator

**Figure 4:**
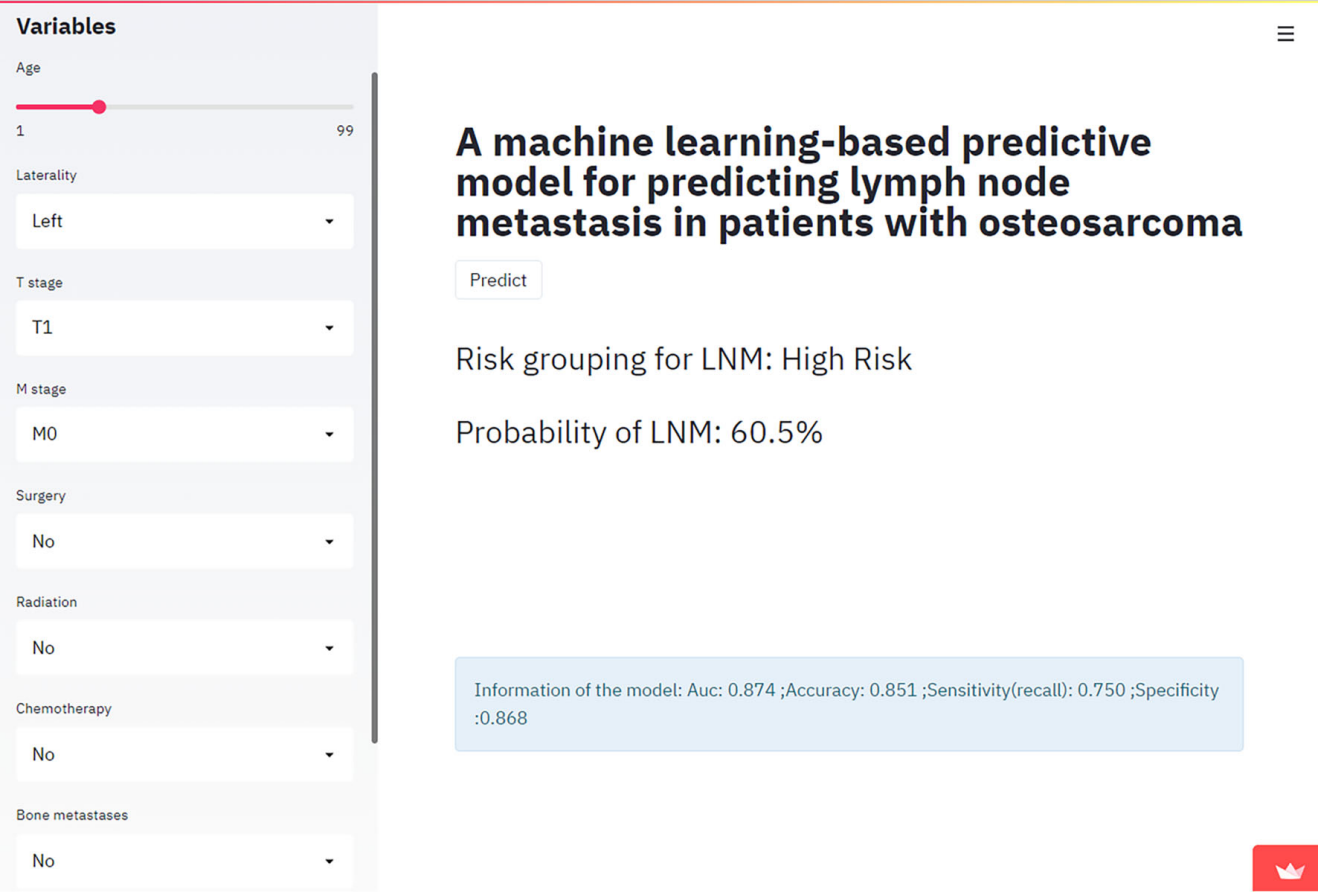
The Web calculator built on the XGB model predicting lymph node metastasis in patients with osteosarcoma.

In this study, an online calculator based on the best model (the XGboost algorithm) was developed to predict the risk of lymph node metastasis in osteosarcoma patients (**Figure 4**). This calculator was easy to automatically present in clinical practice by simply entering the patient’s clinical characteristics and laboratory data. Please refer to the website: https://share.streamlit.io/liuwencai123/os_lnm/main/os_lnm.py.

## Discussion

Osteosarcoma, Ewing sarcoma, and chondrosarcoma were the three most common types of malignant bone tumors. Of the three tumors, osteosarcoma and Ewing sarcoma occur more frequently in childhood, whereas chondrosarcoma is more common in the elderly (18). Pathology is the gold standard for the diagnosis of osteosarcoma, but clinical experience is required to determine the presence of tumor cells, and this empirical variation can influence pathologic judgments (19). It has been shown that there were prognostic differences between patients with different subtypes of osteosarcoma, with a higher overall survival rate for classic osteosarcoma and a lower survival rate for the small cell and capillary dilated types (20). Staging is important in the development of treatment plans. More than 90% of typical osteosarcomas have eroded the bone cortex and invaded soft tissues at the time of consultation (21), and belonged to the interstitial stage IIB type, and those with pulmonary metastases belonged to stage III. The most common site of metastasis in osteosarcoma is the lung (22), but patients with pulmonary metastases have a relatively minor negative prognostic effect compared to metastases from other sites (23). Although the incidence of lymph node metastases from osteosarcoma is low, patients with lymph node metastases from osteosarcoma have a poorer prognosis than those with non-lymph node metastases (9). An animal study showed that the median survival time of dogs without lymph node metastases (318 days; range, 20 to 1,711 days) was significantly longer than the median survival time of dogs with lymph node metastases (59 days; range, 19 to 365 days) (24), and lymph node metastases were an unfavorable prognostic factor. Therefore, early identification of the risk of lymph node metastasis in patients with osteosarcoma is clinically important and will facilitate timely measures by clinicians to optimize treatments.

This study developed and validated several models using popular machine learning algorithms to predict the risk of lymph node metastasis among osteosarcoma patients and the logistic regression analysis showed that T stage, M stage, surgery, and chemotherapy were independent risk factors for lymph node metastasis among osteosarcoma patients. After comparing the performance of the six ML algorithms, we found that the XGB algorithm had the best performance (AUC=0.882). To increase the feasibility of the application of this model, an online web calculator was further developed for assessing the individual probability of lymph node metastasis in patients with osteosarcoma.

The clinical characteristics of age, T stage, laterality, M stage, and surgery were identified by the ML algorithm as the most important predictors of lymph node metastasis in patients with osteosarcoma. The results of this study showed that older patients with osteosarcoma had a greater risk of lymph node metastasis, which may be related to the bimodal nature of the age of predilection for osteosarcoma (25). T stage and M stage, as indicators of the biological progression of the tumor, were positively correlated with lymph node metastasis in a larger number of tumors (26). And surgery as one of the more important variables may cause metastatic dissemination of tumor cells due to invasive operations such as surgery, puncture or intraoperative injury to tissues such as blood vessels (27).

In a study conducted by Dong et al. (28), gender, primary tumor site, tumor type and size were identified as independent risk factors for lymph node metastasis in osteosarcoma by single multifactor analysis, and age, race, distant metastasis, tumor type and surgical treatment were also shown to be prognostic factors affecting overall survival of patients with lymph node metastasis in osteosarcoma by multifactor COX regression analysis. However, despite the significance of the results in this study, no external data validation was performed and there may had been over-fitting. In a large population-based cohort study (29), the presence of bone metastases (OR 8.73; 95%CI: 4.37-17.48) or brain metastases (OR 25.63; 95%CI: 1.55-422.86) in patients with osteosarcoma was significantly associated with the occurrence of pulmonary metastases, which could provide some ideas for our subsequent study to take into account the presence of metastases from other sites before the patient develops lymph node metastases as a factor.

The advantage of the present study is that the ML algorithm was used to develop a prediction model to assess the risk of lymph node metastasis in patients with osteosarcoma using readily available clinical data, and the prediction model developed in this study was validated by the validation group, showing strong predictive power compared with the linear model used in previous studies. The prediction model developed in this study showed strong predictive power and some advantages over the linear models used in previous studies. The inclusion of different ethnic groups in the modeling and validation groups also demonstrated the generalizability of the model. Finally, in order to make the prediction model more convenient for clinical use, an online application based on the model was created, which allowed clinicians to predict the risk of lymph node metastasis in patients with osteosarcoma by using the clinical characteristics of the patients available.

## Limitations

There were some limitations of this study. Firstly, this study was a retrospective study and there might be some selection bias. Secondly, the results analyzed in this study only demonstrated the association between risk factors and lymph node metastasis, but could not elucidate whether there was a causal relationship. Because the original data in the SEER database have no chronological sequences, thus the causal relationship between variables could not be obtained after analysis. Therefore, this study need further investigations.

## Conclusions

This study developed and validated the ML algorithm for individualized prediction of whether a patient with osteosarcoma will develop lymph node metastasis by using readily available clinical features. T stage, M stage, surgery and Chemotherapy are independent risk factors for predicting lymph node metastasis among osteosarcoma patients. Among all the six ML algorithms, the XGB algorithm has the best predictive performance, and the online risk calculator was generated based on this algorithm, which can help clinicians to identify the risk probability of lymph node metastasis among osteosarcoma patients.

## Data Availability Statement

The original contributions presented in the study are included in the article/supplementary material. Further inquiries can be directed to the corresponding authors.

## Ethics Statement

The SEER database is a comprehensive data source developed based on population data and updated annually since its launch in 1973. It is public and identifiably accessible that data analysis is treated as non-human subjects by the Office for Human Research Protections. As such, no institutional review board approval and informed consent were required. For multicenter data, the study was approved by the ethics review committee of four medical institutions in China, the Second Affiliated Hospital of Jilin University, the Second Affiliated Hospital of Dalian Medical University, Liuzhou People’s Hospital, and Xianyang Central Hospital (No. 2021-00-22) and was conducted in accordance with the guidelines of the Helsinki Declaration.

## Author Contributions

CLY, CXG and QL carried out the study design. WCL conducted the research and collected and analyzed the data. WLL and YFL performed the statistical analysis and drafted the manuscript. ZRT and STD provided the expert consultations and suggestions. ALL conceived the study, participated in its design and coordination, and helped shape the language. All authors contributed to the article and approved the submitted version.

## Conflict of Interest

Author CG was employed by Hengpu Yinuo (Beijing) Technology Co., Ltd, Beijing, China.

The remaining authors declare that the research was conducted in the absence of any commercial or financial relationships that could be construed as a potential conflict of interest.

## Publisher’s Note

All claims expressed in this article are solely those of the authors and do not necessarily represent those of their affiliated organizations, or those of the publisher, the editors and the reviewers. Any product that may be evaluated in this article, or claim that may be made by its manufacturer, is not guaranteed or endorsed by the publisher.
